# Exploring the Relationship Between Mental Well-Being, Exercise Routines, and the Intake of Image and Performance Enhancing Drugs During the Coronavirus Disease 2019 Pandemic: A Comparison Across Sport Disciplines

**DOI:** 10.3389/fpsyg.2021.689058

**Published:** 2021-07-06

**Authors:** Mami Shibata, Julius Burkauskas, Artemisa R. Dores, Kei Kobayashi, Sayaka Yoshimura, Pierluigi Simonato, Ilaria De Luca, Dorotea Cicconcelli, Valentina Giorgetti, Irene P. Carvalho, Fernando Barbosa, Cristina Monteiro, Toshiya Murai, Maria A. Gómez-Martínez, Zsolt Demetrovics, Krisztina Edina Ábel, Attila Szabo, Alejandra Rebeca Melero Ventola, Eva Maria Arroyo-Anlló, Ricardo M. Santos-Labrador, Inga Griskova-Bulanova, Aiste Pranckeviciene, Giuseppe Bersani, Hironobu Fujiwara, Ornella Corazza

**Affiliations:** ^1^Department of Neuropsychiatry, Graduate School of Medicine, University of Kyoto, Kyoto, Japan; ^2^Laboratory of Behavioral Medicine, Neuroscience Institute, Lithuanian University of Health Sciences, Palanga, Lithuania; ^3^Laboratory of Neuropsychophysiology, Faculty of Psychology and Education Sciences, University of Porto, Porto, Portugal; ^4^School of Health, Polytechnic of Porto, Porto, Portugal; ^5^Department of Neurodevelopmental Psychiatry, Habilitation and Rehabilitation, Graduate School of Medicine, Kyoto University, Kyoto, Japan; ^6^Organization for Promotion of Neurodevelopmental Disorder Research, Kyoto, Japan; ^7^Department of Clinical, Pharmaceutical and Biological Sciences, School of Life and Medical Sciences, University of Hertfordshire, Hatfield, United Kingdom; ^8^Clinical Neurosciences and Mental Health Department and CINTESIS, Faculty of Medicine, University of Porto, Porto, Portugal; ^9^Department of Psychometrics, Institute of Psychology, Federal University of Rio de Janeiro, Rio de Janeiro, Brazil; ^10^Department of Psychology, Pontifical University of Salamanca, Salamanca, Spain; ^11^Institute of Psychology, ELTE Eötvös Loránd University, Budapest, Hungary; ^12^Centre of Excellence in Responsible Gaming, University of Gibraltar, Gibraltar, Gibraltar; ^13^Institute of Health Promotion and Sport Sciences, ELTE Eötvös Loránd University, Budapest, Hungary; ^14^Faculty of Psychology, Pontifical University of Salamanca, Salamanca, Spain; ^15^Department of Psychobiology, Neuroscience Institute of Castilla-León, University of Salamanca, Salamanca, Spain; ^16^Escuela Universitaria de Magisterio Fray Luis de León, Valladolid, Spain; ^17^Department of Neurobiology and Biophysics, Institute of Biosciences, Vilnius University, Vilnius, Lithuania; ^18^Department of Medico-Surgical Sciences and Biotechnologies, Sapienza University of Rome, Rome, Italy; ^19^Artificial Intelligence Ethics and Society Team, RIKEN Center for Advanced Intelligence Project, Saitama, Japan

**Keywords:** excessive exercise, supplement, IPEDS, COVID-19, enhancement

## Abstract

**Introduction:** Physical distancing under the coronavirus disease 2019 (COVID-19) pandemic had a significant impact on lifestyles, including exercise routines. In this study, we examined the relationship between mental health and addictive behaviors, such as excessive exercise and the use of image and performance enhancing drugs (IPEDs) across 12 sport disciplines.

**Materials and methods:** A large cross-sectional sample of the adult population (*N* = 2,295) was surveyed. The mean age was 33.09 (*SD* = 11.40). The number of male participants was 668 (30.0%). The use of IPEDs was assessed in conjunction with psychometric measures such as the Exercise Addiction Inventory (EAI) and the Appearance Anxiety Inventory (AAI). The participants were grouped into activity group (AG) and non-activity group (NAG) according to the presence or absence of their exercise habits. The results were compared between these groups, as well as across sport disciplines, while taking into account the relationship between different psychological measures and IPEDs consumption.

**Results:** The frequency of IPEDs use was higher among AG (34.6%) than NAG (14.6%), although AG participants reported less history of addictions (7.1%) than NAG (11.8%). The logistic regression analysis revealed that scores equal to or above cutoff points, in both the EAI and AAI, predicted the IPEDs use. Regarding the differences across the various sport disciplines, those who were involved in practicing Weight Lifting and Cross Fit were found to be more at risk of excessive exercising and more inclined to use a wide range of IPEDs.

**Conclusions:** Although exercise could help to increase well-being and prevent addictions during the COVID-19 pandemic, our results show that those in the AG are particularly vulnerable to excessive IPEDs use. Sport disciplines associated with higher EAI and AAI scores have also shown a higher tendency to excessive IPEDs use. Furthermore, the factor of having above the cutoff scores in EAI or AAI in each sport could indicate larger IPEDs consumption regardless of the discipline. In light of the current findings, it is necessary to better define the “non-excessive” levels of exercise in various sport disciplines and an adequate intake of IPEDs to ensure the safety and well-being of people during a pandemic.

## Introduction

The coronavirus disease 2019 (COVID-19) pandemic has posed a sudden and unprecedented challenge to public health, leading to dramatic lifestyle changes (Basu et al., 2020; Di Renzo et al., 2020). In this context, exercise could play an important part as a coping strategy to deal with stressful situations. Evidence suggests that regular exercising could have a positive impact on mental health, improving depressive mood, anxiety (Huang et al., 2015; Schuch et al., 2015; Stonerock et al., 2015; Callow et al., 2020; Herbert et al., 2020), and psychological-related issues (Hassmén et al., 2000; Schuch et al., 2016). It also helps to improve cognitive and affective functions, such as memory (Parfitt et al., 2000; Suwabe et al., 2018) and attention (de Sousa et al., 2019). Conversely, excessive exercise, known as “compulsive exercise,” “problematic exercise,” or “exercise addiction,” can have a negative influence on mental and physical well-being (Berczik et al., 2012; Kreher and Schwartz, 2012). Although excessive exercise has not been formally defined as an addiction in the 5th edition of the Diagnostic & Statistical Manual of Mental Disorders (DSM-5; American Psychatric Association, 2013), the concept has been increasingly debated as part of a wide range of other behavioral, non-drug-related forms of addiction, such as gambling, Internet use, gaming, eating/food, sex, and shopping, possibly leading to damaging health consequences, especially among vulnerable groups (Banz et al., 2016; Fineberg et al., 2018; Lichtenstein et al., 2018; Petry et al., 2018).

It is known that exercise and the use of drugs, such as image and performance enhancing drugs (IPEDs), or licit boosters (e.g., supplements, medicinal products), often coexist as a way to better achieve the ultimate goal in a sport discipline (Peeling et al., 2018). The concept of “enhancement” is defined here as an “intervention aimed to improve the human form and function more than necessary for maintaining health or recovery for purposes other than treating a disease” (Caplan and Elliott, 2004; Juengst Eric, 2004). Previous studies have shown that IPEDs are increasingly bought over the Internet without any medical supervision, possibly leading to excessive or even toxic intakes (Mooney et al., 2017; Corazza et al., 2019). For example, the overuse of caffeine tablets could induce negative outcomes, such as insomnia or sudden death (Ronis et al., 2018; Booth et al., 2020; Sweeney et al., 2020), with caffeine use disorder being one of the conditions mentioned in the DSM-5 under a section on “Conditions for Future Study” (Ágoston et al., 2018). It is worth noting that in some cases, licit supplements themselves can contain undisclosed prohibited substances by the World Anti-Doping Agency (WADA) and thus expose users to unwanted health risks (Mazzoni et al., 2017; Helle et al., 2019). Regarding the relationship between excessive physical exercise and IPEDs use, Corazza et al. (2019) indicated that the risk of excessive exercising is positively correlated with IPEDs use. However, both are beneficial to health at low to intermediate levels.

When the COVID-19 pandemic was officially recognized by the World Health Organization (WHO) in March 2020, it was assumed that the engagement with certain rewarding behaviors, as represented by physical exercise during a period when there was no access to gyms, would have increased considerably as a coping strategy during prolonged periods of self-isolation. Although exercise might have helped to alleviate stress and difficult thoughts at such challenging times, it might also have contributed to a reduced engagement in social interactions and other daily activities, simultaneously facilitating the excessive intake of IPEDs and the development of other risky habits that are then difficult to break. The diffuse advertising of IPEDs on social media and other online platforms (Kishimoto et al., 2009; Dubey et al., 2020; King et al., 2020; Sun et al., 2020) might have made their consumption even more likely when individuals spent more time on the Internet and initiated the consumption among new vulnerable cohorts (Bhatti et al., 2020). Supporting evidence emerged from a recent study on the use of IPEDs among the general adult population (*N* = 3,161) during the COVID-19 pandemic. It was found that 28% of the participants reported use of such products with 6.4% of them starting a new use during the pandemic (Dores et al., 2021). Interestingly, the only predictor of change in IPEDs consumption was the score on or above the cut-off point on the Exercise Addiction Inventory (EAI) (OR = 2.272), indicating that excessive exercising could potentially induce excessive IPEDs use worldwide (Dores et al., 2021).

However, to date, the differences among sport disciplines in the occurrence of excessive exercising and their association with IPEDs use during the pandemic are still unknown, and this is the first study that investigates these issues. In this context, we hypothesized that the engagement with exercise and IPEDs use as a coping strategy during the pandemic might have changed significantly across the various sport disciplines. Each specialty is, in fact, unique in nature, and endurance athletes, such as ball game players, fitness center attendees, and those engaged in power disciplines, have already shown a higher risk of excessive exercising in a pre-pandemic situation (Di Lodovico et al., 2019). We were also interested in assessing the relationship between excessive exercise and different types of psychological functioning, such as appearance anxiety (Corazza et al., 2019; Trott et al., 2020) and self-compassion (Neff, 2003), and their differences among various sports. While higher scores in terms of the Appearance Anxiety Inventory (AAI) might have been indicative of the individuals being more concerned and critical about their physical appearance during the lockdown, higher scores on the Self-Compassion Scale (SCS), a concept that closely overlaps with mindfulness and Zen Buddhism (Barczak and Eklund, 2020), might have been indicative of the role played by “mind–body” training (Nakao and Ohara, 2014), in developing a safe and a positive attitude toward a challenging situation and in acting as a mitigating factor toward excessive exercise and IPEDs use. For instance, it has been previously found that those who were involved in practicing intensive mind–body training, such as martial arts players, aimed at achieving a deeper integration between mind (psychology) and body (exercise) (Nakao and Ohara, 2014)and reported a number of cognitive health benefits as a result of their training (Fujiwara et al., 2019).

More specifically, in this study, we aimed to investigate the differences in excessive exercising, appearance anxiety, and self-compassion as a related psychological measure between (a) subjects engaged with habitual exercise routines (activity group, AG) and non-exercisers (non-activity group, NAG) and (b) across different sport disciplines. We hypothesized that the scores of AAI and SCS would be significantly higher in the AG. As for comparisons among different sport disciplines, we hypothesized that several disciplines that demand relatively high-intensity functional training would show a higher level of EAI and AAI.

Besides, we aimed to identify the relevance of IPEDs use in association with abovementioned psychological measures. In this regard, we expected to find a more diffuse use of IPEDs in AG than in NAG. Finally, we examined the relationships between psychological measures, including excessive exercising, and IPEDs use in both AG and NAG across various sport disciplines.

## Materials and Methods

### Participants

Overall, 2,295 participants were included in the survey. They were from Brazil (*n* = 737; 32.1%), Italy (*n* = 647; 28.2%), Spain (*n* = 264; 11.5%), Lithuania (*n* = 224; 9.8%), Portugal (*n* = 177; 7.7%), the United Kingdom (*n* = 129; 5.6%), Japan (*n* = 70; 3.1%), and Hungary (*n* = 47; 2.0%). The mean age of the sample was 33.09 (*SD* = 11.40) with a greater proportion of female (*n* = 1,607; 70.0%) over male (*n* = 688; 30.0%) participants.

The AG corresponded to the participants who practiced more than one sport in this investigation. The AG comprised 87.5% (*n* = 2,007) of the respondents. The remaining 12.5% (*n* = 288) were included in the NAG. Age difference was not found between the AG and NAG [AG/NAG, 33.13 ± 11.54/32.80 ± 10.39, *t*_(2293)_ = −0.47; *p* = 0.642].

As shown in Table 1, they were engaged in a variety of sports, mainly Generic Workout (*n* = 769; 38.3%), Walking (*n* = 387; 19.3%), Weight Lifting (*n* = 355; 17.7%), Running (*n* = 301; 15.0%), Yoga (*n* = 253; 12.6%), Fighting Sports (e.g., Boxing, Kick boxing, and Martial arts) (*n* = 146; 7.3%), Swimming (*n* = 135; 6.7%), Dance (*n* = 128; 6.4%), Martial Arts (*n* = 109; 5.4%), Cycling (*n* = 99; 4.9%), Ball Sports (*n* = 73; 3.6%), Budo (*n* = 67; 3.3%), and Cross Fit (*n* = 63; 3.1%). For clarity reasons, “Generic Workout” included subjects who were engaged with some general running, Weight Lifting, and other free-body exercises to keep fit and tone the muscles; “Martial Arts” meant oriental (non-Western cultural style) fighting sports; “Budo” corresponded to Japanese-origin martial arts, such as Kendo, Aikido, Judo, and Karate.

**Table 1:** Type of activities (*N* = 2,295).

Generic workout	38.3%
Walking	19.3%
Weight lifting	17.7%
Running	15.0%
Yoga	12.6%
Fighting sports	7.3%
Swimming	6.7%
Dance	6.4%
Martial arts	5.4%
Cycling	4.9%
Ball sports	3.6%
Budo	3.3%
Cross fit	3.1%
Mountain	2.9%
Tennis	1.7%
Triathlon	0%
Other	3.7%
None activity	12.5%

*Generic Workout: some general running, Weight Lifting, and other free-body exercises to keep fit and tone the muscles*.

*Martial Arts: oriental (non-Western cultural style) fighting sports, such as Kendo, Judo, Aikido, Karate, Taekwondo, Brazialian jiu-jitsu, Muay Thai, Wushu, Tai Chi, and Capoeira*.

*Budo: Japanese-origin martial arts, such as Kendo, Judo, Aikido, and Karate*.

### Instruments

The survey of this study covered the following:

a) demographic information (age, gender, presence of habitual exercise, and style of sports);
b) levels of excessive exercising, appearance anxiety, and self-compassion;
c) IPEDs use to enhance fitness performance or to make the appearance of individuals look good before/after physical distancing. The questions were in the multiple choice format, and the list of choices consisted of vitamins, green tea extract, and whey protein (for details, see Supplementary Table 1.1–1.4);
d) history of addictions and any worsening during physical distancing;
e) changes in the amount of drinking and smoking during physical distancing.

### Exercise Addiction Inventory

We used the EAI to assess the risk of excessive exercising (Terry et al., 2004). The EAI contains six items rated on a five-point scale (e.g., “Exercise is the most important thing in my life”). The total score range is from 6 to 30 with a score of ≥24 being indicative of exercise addiction. The higher scores indicate a higher risk of exercise addiction. The internal reliability of the EAI for the total sample was acceptable (Cronbach's α = 0.685), and the scale has been validated in various nations/translations (Griffiths et al., 2015).

### Appearance Anxiety Inventory

The AAI was used to assess the degree of cognitive processes and behavior characteristics of body dysmorphic disorder (BDD) (Veale et al., 2014). The AAI is a 10-item questionnaire rated on a 5-point Likert scale (e.g., I check my appearance in mirrors by touching with my fingers or taking photos of myself). The total score range is 0–40 with a cutoff score of ≥21 being indicative of a risk of BDD. The higher scores suggest a higher risk of appearance anxiety/BDD. The internal reliability was good (Cronbach's α = 0.87).

### Self-Compassion Scale-Short Form

The higher scores of SCS indicate more compassion toward oneself, namely, kindness to self. We used the SCS-Short Form (SCS-SF) in this study (Raes et al., 2011). This scale has 12 items reflecting on attitudes toward oneself (e.g., “when something painful happens, I try to take a balanced view of the situation”), resulting in its total scores ranging from 12 to 60. A high internal reliability was observed for the total sample (Cronbach's α = 0.82), and the questionnaire has been translated in different languages (Dores et al., 2021).

### Procedures

This study was approved by the University of Hertfordshire Health and Human Science Ethics Committee with Delegated Authority (ECDA) [aHSK/SF/UH/00104(2)] and by the Ethics Committees of all the participating organizations. This study was conducted according to the Declaration of Helsinki (World Medical Asociation, 2018). All responses were anonymous, securely stored, and made accessible only to the members of the research team.

A study questionnaire on “Fitness Lifestyle, Body Image and New Habits During the Corona Virus Pandemic (COVID-19)” was disseminated at the start of the pandemic from April to June in 2020 *via* the web-based platform Qualtrics (Fisher, 2020). Dissemination activities were supported by established laboratories based in eight countries (the United Kingdom, Italy, Lithuania, Hungary, Portugal, Spain, Japan, and Brazil), and anonymized data were stored in a secure platform at the University of Hertfordshire. The questionnaires were translated and back-translated from English into Italian, Spanish, Japanese, Portuguese, Hungarian, and Lithuanian. This study involved participants aged 18 years and above from a broad spectrum of the general population. A “snowball sampling” technique was employed. It was advertised widely on social media platforms, including Facebook groups (e.g., Fitness and Well-being groups), LinkedIn, WhatsApp, Twitter, Instagram, and also on sport-related websites/groups. There were no exclusion criteria. All participants gave written informed consent to participate.

### Statistical Analysis

The data were analyzed with SPSS Version 22.0.0.0 (IBM, USA). The *t*-test and ANOVA were used to examine the continuous variable, and chi-square tests were used to test the categorical variables.

#### Group Comparison Between Activity Group vs. Non-activity Group

The differences reported by regular exercisers (AG) and non-exercisers (NAG) were assessed. The *t*-tests were applied to compare the EAI, AAI, and SCS scores, and chi-square tests were used for the comparisons in terms of IPEDs use, change in the amount of smoking and drinking, and the presence of any addiction history before and after physical distancing. In the chi-square tests, Bonferroni corrections were applied for multiple comparisons of data related to changes in psycho-behavioral measures before and after the COVID-19 pandemic, resulting in a statistical significance *p* = 0.05/6 = 0.0083 (the six items used were the use of IPEDs, initiation of IPEDs use during physical distancing, increase of smoking during physical distancing, increase of drinking during physical distancing, history of addiction, and worsening the addiction problem during physical distancing) for each sport discipline.

#### Group Comparison Among Different Kinds of Sport Disciplines

The ANOVA analysis was carried out to compare the mean EAI, AAI, and SCS scores among sport disciplines in which the number of the players was above 3% of all subjects. The Tamhane's T2 method was used for *post-hoc* multiple comparisons as the results indicated no equal variances (i.e., heteroscedasticity) among various sport disciplines for the EAI and AAI scores. With equal variances in the SCS scores, the Tukey's *post-hoc* procedure was performed for multiple comparisons. In addition, chi-square tests were used for the comparisons regarding the presence of IPEDs use, change in the amount of smoking and drinking, and any addiction history before and after physical distancing. The comparisons were performed between one of the disciplines and others. The Bonferroni corrections were applied for multiple comparisons of the data related to the changes in psycho-behavioral measures before and after the COVID-19 pandemic after chi-square tests (i.e., the statistical significance *p*-values were set at 0.05/5 = 0.01, and the five items were the use of IPEDs, increase of smoking during physical distancing, an increase of drinking during physical distancing, history of addiction, and worsening the addiction problem during physical distancing).

#### Logistic Regression Between Psychological Indices (EAI, AAI, and SCS) and IPEDs Use

The logistic regression analyses were performed to investigate how the EAI, AAI, and SCS predict the IPEDs use in both the AG and the NAG and across the investigated sport disciplines. The response variable was the usage of IPEDs (classified as 0, “not used” or 1, “used”), while the explanatory variables were age, gender (classified as 1, “male participants” or 2, “female participants”), equal to or above cutoff points of the EAI and AAI (classified as 0, “under the cutoff points” or 1, “equal or above cutoff points”), and total scores of the SCS.

## Results

### Psychological Measures

The average scores for the whole samples were as follows: *M* = 16.52 and *SD* = 4.16 (EAI); *M* = 16.84 and *SD* = 5.55 (AAI); *M* = 30.9 and *SD* = 6.02 (SCS); and 736 (32.1%) individuals used IPEDs. The numbers of those who scored above the cutoff points of EAI or AAI were 96 (4.2%) (EAI ≥24) and 480 (20.9%) (AAI ≥ 21).

### Addiction and Increase of Smoking and Drinking

History of addictions was reported among 7.7% (*n* = 176) of the participants with most of the sample (*n* = 2,119; 92.3%) having no previous addiction history (Table 2). The habitual smokers [(*n* = 471; 20.5%), 37.2% (*n* = 175)] felt the need to smoke more during physical distancing, while habitual drinkers [(*n* = 1934; 84.3%), 16.8% (*n* = 325)] felt the need to increase the consumption of alcohol during physical distancing.

**Table 2:** Rate of addiction, an increase of smoking and drinking (*N* = 2,295).

**History of addiction**	***N* (%)**
Yes	176 (7.7%)
No	2,119 (92.3%)
Increase during	49 (27.8%)
**Increase of smoking**	***N*** **(%)**
No increase	296 (62.8%)
Increase	175 (37.2%)
**Increase of drinking**	***N*** **(%)**
No increase	1,609 (83.2%)
Increase	325 (16.8%)

### Group Comparison Between Activity Group vs. Non-Activity Group and Among Different Kind of Sport Disciplines

**Figure 1:**
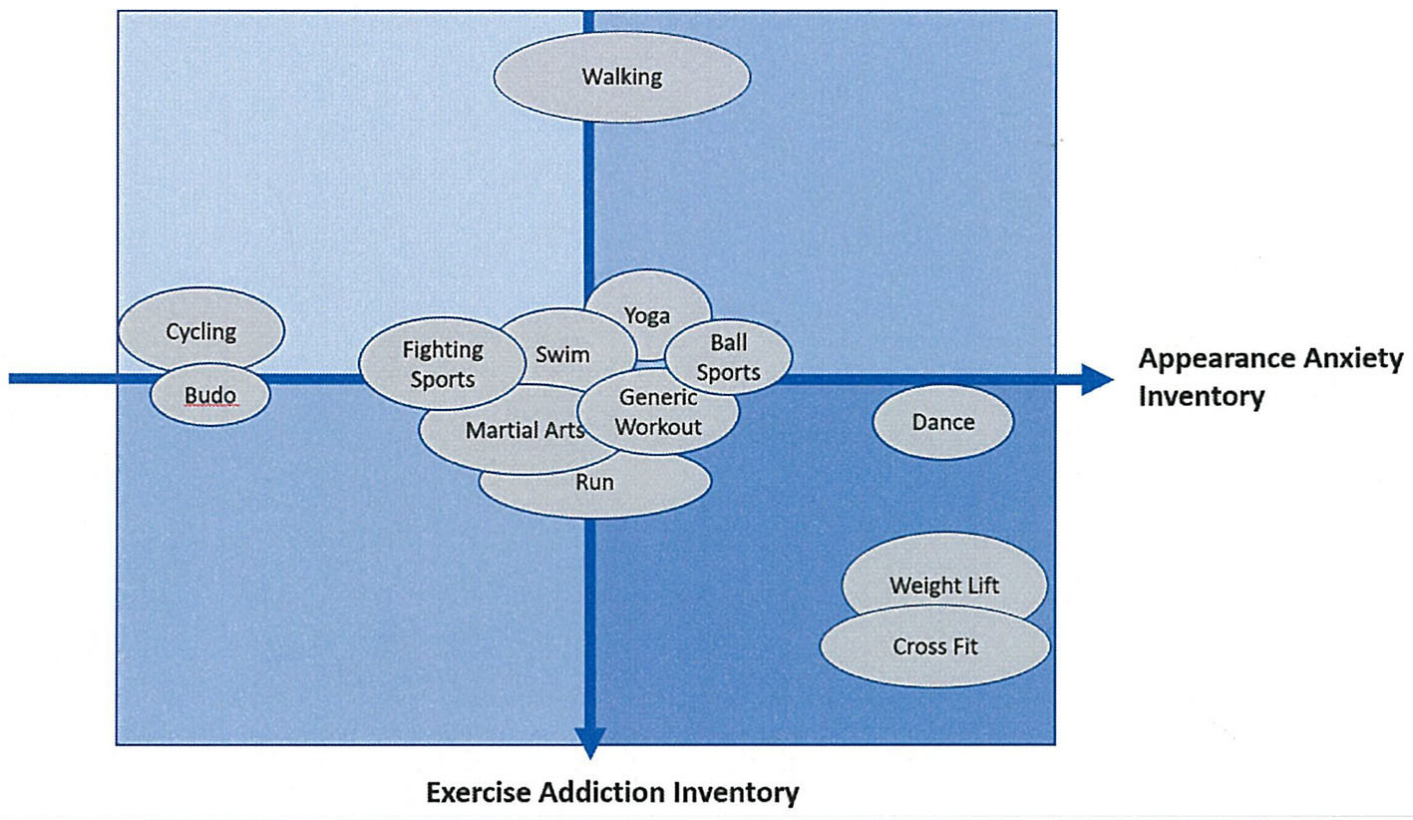
Schematic representation of the features of EAI and AAI on each exercise.

As shown in Table 3, rate of IPEDs users was significantly higher in AG than NAG (AG/NAG, *N* = 694; 34.6% / *N= 42*; 14.6%, χ^2^ (2, *n* = 736) = 46.22;*p* < 0.001). Rate of participants who had history of addiction was lower in AG than NAG (AG/NAG, *N* = 142; 7.1% / *N* = 34; 11.8%, χ^2^(2, *n* = 176) = 7.96;*p* = 0.005). The group comparisons between the different sport disciplines are shown in Supplementary Tables 2–4 and schematically summarized in Figure 1. The differences within the groups of different sport disciplines, including NAG, were also observed for EAI, *F*_(15, 3032)_ = 8.11 and *p* < 0.001. The Tamhane's T2 method-based *post-hoc* analysis showed the EAI in walking to be considerably lower compared with other sport disciplines (*p* < 0.009), while Weight Lifting (*p* < 0.02) and Cross Fit (*p* < 0.03) had higher EAI scores compared with other sport disciplines. The differences within the groups of sport disciplines including NAG were also observed for AAI, *F*_(16, 3319)_ = 6.98 and *p* < 0.001. For this group, the Tamhane's T2 test revealed Budo (*p* < 0.02) and Cycling (*p* < 0.03) AAI scores to be lower compared with other sport disciplines, while Weight Lifting (*p* < 0.05), Cross Fit (*p* < 0.04), and Dance (*p* < 0.04) reached higher AAI scores compared with the other sport disciplines.

**Table 3:** Differences in IPEDs use and behavioral changes between AG and NAG.

	**NAG**	**AG**	
Usage of IPEDs	42 (14.6%)	694 (34.6%)	***x***^**2**^ **=** **46.22;** ***p*** **<** **0.001**
Start of IPEDs during physical distancing	10 (3.9%)	102 (7.2%)	***x***^2^ = 3.78; *p* = 0.052
Increase of smoking during physical distancing	52 (47.7%)	123 (34.0%)	***x***^2^ = 6.76; *p* =0.009
Increase of drinking during physical distancing	28 (9.7%)	297 (14.8%)	***x***^2^ = 5.34; *p* = 0.021
History of addiction	34 (11.8%)	142 (7.1%)	***x***^**2**^ **=** **7.96;** ***p*** **=** **0.005**
Worsening the addiction problem during physical distancing	7 (20.6%)	42 (29.6%)	***x***^**2**^ = 1.10; *p* = 0.294

*AG, activity group; NAG, non-activity group*.

*According to Bonferroni correction, all p < 0.0083 should be considered significant*.

There was a tendency for SCS scores to differ across sport disciplines including NAG, *F*_(16, 3319)_ = 1.64 and *p* = 0.052.

Some sports showed significantly higher, or lower, percentages in IPEDs user than other disciplines. More specifically, IPEDs use was significantly higher in Weight Lifting [(*n* = 217; 61.1%), χ^2^(2, *n* = 694) = 134.37, and *p* < 0.001] and in Cross Fit [(*n* = 38; 60.3%), χ^2^(2, *n* = 694) = 19.047, and *p* < 0.001], while it was significantly lower in Walking [(*n* = 95; 24.5%), χ^2^(2, *n* = 694) = 21.33, and *p* < 0.001], than in other disciplines. The rate of subjects who had history of addiction and subjects who experienced worsening of it during physical distancing did not differ among all disciplines. The rate of those who had increased their smoking was higher in Fighting Sports [(*n* = 13; 61.9%), χ^2^(2, *n* = 123) = 7.75, and *p* = 0.005], but lower in Weight Lifting [(*n* = 9; 17.3%), χ^2^(2, *n* = 123) = 7.52, and *p* = 0.006], and those who had increased their drinking was lower in Generic Workout [(*n* = 92, 12.0%), χ^2^(2, *n* = 297) = 7.95, and *p* = 0.005].

### Logistic Regressions Between EAI, AAI, and SCS, and IPEDs Use

The logistic regression analyses revealed that being equal to or above cutoff points of the EAI predicted the IPEDs use in the AG (OR = 2.226, CI 95% [1.438–3.444], and *p* = 0.000) (Supplementary Table 5). Being equal to or above cutoff points of the AAI also predicted the IPEDs use in the AG (OR = 2.009, CI 95% [1.571–2.571], and *p* = 0.000). Female gender also predicted the IPEDs use in the AG (OR= 0.614, CI 95% [0.502–0.750], and *p* < 0.001).

Regarding the analyses of each kind of discipline, being equal to or above cutoff points of the EAI predicted the IPEDs use in Generic Workout (OR = 2.047, CI 95% [1.013–4.135], and *p* = 0.046), Yoga (OR = 9.805, CI 95% [1.969–48.824], and *p* = 0.005), and Fighting Sports (OR = 12.984, CI 95% [1.461–115.410], and *p* = 0.021). Being equal to or above cutoff points of the AAI also predicted the IPEDs use in Generic Workout (OR = 1.604, CI 95% [1.066–2.413], and *p* =0.023), Walking (OR = 2.214, CI 95% [1.166–4.201], and *p* = 0.015), Weight Lifting (OR = 2.104, CI 95% [1.210–3.658], and *p* = 0.008), Running (OR = 2.061, CI 95% [1.070–3.974], and *p* = 0.031), Fighting Sports (OR = 3.219, CI 95% [1.144–9.058], and *p* = 0.027), and Dance (OR = 2.872, CI 95% [1.096–7.521], and *p* = 0.032). The SCS scores did not predict the IPEDs use in any discipline. Female gender also predicted the IPEDs use in Generic Workout (OR = 0.391, CI 95% [0.275–0.555], and *p* = 0.000) and Weight Lifting (OR = 0.631, CI 95% [0.401–0.993], and *p* = 0.047).

## Discussion

Although excessive exercising has been previously correlated with excessive IPEDs use during the COVID-19 physical distancing (Dores et al., 2021), this is the first study assessing the differences across various sport disciplines in terms of EAI, AAI, and SCS and their level of engagement in physical activity (AG vs. NAG).

Overall, we found that the AG group reported a higher IPEDs use, but was less affected by the history of addictions compared with the NAG. In addition, the group comparison between AG and NAG revealed that neither the mean scores of AAI nor SCS differ in AG from those in NAG. These results could support the findings of a previous study that AAI and SCS were non-significant predictors of the habit of physical exercise (Dores et al., 2021), while less history of addictions in the AG indicates that habitual exercise may have health benefits in preventing addictions in general.

The results of the EAI and the AAI varied significantly among various sport disciplines (Supplementary Tables 2, 3). As shown in Figure 1, we categorized the sports into three groups according to the levels of EAI: (1) high EAI group, (2) low EAI group, and (3) others. Weight Lifting and Cross-Fit corresponded to the high EAI group. Only Walking corresponded to the low EAI group. These results confirm the previous evidence (Di Lodovico et al., 2019), according to which endurance athletes, ball game players, fitness center attendees, and those engaged in power disciplines have a high risk of excessive exercising. It is worth noting that in those reporting a high score in the EAI, Weight Lifting and Cross Fit also showed a higher IPEDs use. Conversely, a sport discipline in the low EAI group, that is, Walking, was significantly associated with a lower rate in IPEDs use. Those who perform Weight Lifting often pursue strength and muscle hypertrophy. However, when the training becomes obsessive and compulsive, it could lead to Muscle Dysmorphia (Maida and Armstrong, 2005; Mosley, 2009). Since Cross Fit is recognized as a kind of high-intensity functional training with a high risk of excessive exercise (Lichtenstein and Jensen, 2016; Claudino et al., 2018), this sport could have some traits in common with Weight Lifting. Consequently, it could be suggested that “the higher the EAI, the higher the IPEDs use” indicates that excessive exercising is associated with the risk of cross-addiction with another substance intake. Actually, it was proposed that 15% of those at-risk for excessive exercising may have co-occurring substance addictions, such as nicotine and alcohol addictions (Sussman et al., 2011), although this was not confirmed by other investigations (Szabo et al., 2018). Still, the fact that 12.2% of our sample purchased IPEDs over the Internet might be indicative of a cross-addiction in some individuals, especially when exposed to excessive Internet use during the COVID-19 pandemic (Sun et al., 2020). In other words, the co-occurrence of IPEDs and excessive Internet use during the COVID-19 pandemic has forced individuals to change their lifestyle drastically, leading in some cases to the development of new risky behaviors, which might then be difficult to break.

Some interesting results also emerged in terms of self-compassion, which has been previously associated with high coping skills and motivation of athletes toward their training in sports (Barczak and Eklund, 2020). The high level of self-compassion has also inversely been related to the risk of a substance use disorder, such as alcohol and cannabis (Phelps et al., 2018; Wisner and Khoury, 2020). In this study, the case of Cycling was of particular relevance, in which this sport discipline reported higher SCS and lower AAI scores. Although more studies across different levels of training are required, the current findings might suggest that high self-compassion might have contributed to lower AAI and adequate engagement in Cycling. As a suggestion, more targeted “mind–body” training programs, involving the cultivation of self-compassion, could play an important role in improving coping skills or reducing the risk of substance use disorder and act as a meaningful addiction.

Regarding the finding in logistic regressions, a higher rate in IPEDs use in AG, together with a significant prediction of IPEDs use by being equal to or above cutoff level of EAI and AAI in AG, indicates potential risks of exercise for IPEDs use. Furthermore, we found that the score of EAI and AAI above the cutoff level predicted the IPEDs use in AG. This finding is in line with the previous report before the occurrence of the COVID-19 pandemic (Corazza et al., 2019). Pathological levels of excessive exercising and appearance anxiety could be risk factors for excessive IPEDs use, indicating that it would be meaningful for individuals with high EAI and/or AAI to be aware of the risks of the “excessive enhancement” by IPEDs use. They would have to pay attention to the strategies of approaching each activity, such as checking the risk of burnout and compulsiveness with the exercises from subjective, together with objective points of view (Kreher and Schwartz, 2012; Weinstein and Weinstein, 2014). Also, the logistic regression analysis revealed that being equal to or above cutoff points of EAI or AAI predicted the IPEDs use in 7 out of 13 kinds of disciplines. A possible interpretation is that those who have an apparent tendency for either excessive exercise or appearance anxiety could have a high risk of excessive IPEDs use, regardless of what discipline of exercise people are engaged in.

There are several limitations to this study. First, the number of subjects scoring equal or above the cutoff points of EAI and AAI was small. Therefore, further research would be needed to further clarify the relationship between excessive exercising and IPEDs use in larger samples. Second, the internal reliability of the EAI is low, but we adopted that measure because this study was exploratory. Third, the changes of the EAI and AAI before, during, and after the pandemic were not examined in this study. Therefore, causal relationships among these measures were still unclear. A longitudinal study would be expected to be performed during the time course, i.e., before, during, and after this pandemic. Fourth, the interpretations of the results should be cautious because of the discrepancy in gender valance. Finally, the types of sport disciplines should be expanded, for example, dividing ball sports into specific disciplines, such as baseball, football, and table tennis, in order to clarify the characteristics of each discipline more precisely, such as the tendency of falling into excessive exercise or IPEDs use.

While for mental health benefits of exercise in general, this study showed how excessive exercising could potentially be associated with excessive IPEDs use, which may contribute to further negative outcomes during the COVID-19 pandemic. It is worthwhile paying attention to the risks associated with IPEDs consumption, particularly in individuals with traits of excessive exercising, or appearance anxiety, especially in disciplines that demand high-intensity functional training, who emerged to be most at risk. At the same time, those who are more vulnerable to develop excessive exercising patterns, or manifest appearance anxiety, could be more prompt to use IPEDs regardless of the discipline of practice. As the global COVID-19 pandemic persists, longitudinal studies would be needed to examine the causal relationship between exercise habits and IPEDs use for a longer period of time.

Overall, our study sheds new light on the relatively new concept of “excessive exercising” and its association with IPEDs use and related psychological measures among different sport disciplines at the start of the COVID-19 pandemic, which has influenced our daily habits dramatically. Findings emerging from our work suggest the need for more balanced mental health promotion strategies in terms of both positive and negative aspects of habitual exercise and their associations with other mental health conditions, particularly in regard to the overlooked cross-addiction between excessive exercising and overuse of substances, including IPEDs. It also has a strong relevance in terms of doping prevention as we were able to identify the diffuse intake of IPEDs among amateur athletes of defined sport disciplines, thus contributing to “clean and fair play” according to the definition of anti-doping provided by WADA. Finally, it contributes to a more informed discussion on what constitutes “good exercise habits” in terms of health benefits among various exercise forms during exceptional circumstances such as the current COVID-19 pandemic. Because of the implementation of physical distancing measures, the lifestyles and the coping strategies adopted by people with addiction have drastically changed. While face-to-face services have played an important role in supporting these individuals before the pandemic, they might now consider alternative forms of intervention, such as telephone and virtual reality-based programs which have been developed during the pandemic (Liese and Monley, 2021). Excessive exercising (and the related excessive IPEDs consumption) may be prevented by these newly established strategies for health services.

## Data Availability Statement

The raw data supporting the conclusions of this article will be made available by the authors, without undue reservation.

## Ethics Statement

The studies involving human participants were reviewed and approved by University of Hertfordshire Health and Human Science Ethics Committee with Delegated Authority. Written informed consent to participate in this study was provided by the participants' legal guardian/next of kin.

## Author Contributions

HF and OC: conceptualization and resources. HF, OC, and ZD: funding acquisition. HF, OC, and MS: methodology. JB: formal analysis. MS, JB, AD, KK, PS, ID, DC, VG, IC, FB, CM, TM, MG-M, ZD, KÁ, AS, AV, EA-A, RS-L, IG-B, AP, GB, and HF: investigation and data collection. HF, MS, JB, and OC: data curation. MS: writing—original draft preparation. KK, SY, AD, FB, ZD, AS, ID, HF, and OC: writing—review and editing. MS and KK: visualization. MS, HF, and OC: supervision. OC: project administration. All authors have read and agreed to the published version of the manuscript.

## Conflict of Interest

The authors declare that the research was conducted in the absence of any commercial or financial relationships that could be construed as a potential conflict of interest.
