# Supplementary material for: Exploring the Relationship Between Mental Well-Being, Exercise Routines, and the Intake of Image and Performance Enhancing Drugs During the Coronavirus Disease 2019 Pandemic: A Comparison Across Sport Disciplines
Source: Front Psychol. 2021 Jul 6;12:689058. doi: 10.3389/fpsyg.2021.689058 (PMC8290335; doi:10.3389/fpsyg.2021.689058)
Supplement: Supplementary file 1 [file Data_Sheet_1.PDF]

Supplementary Material

1 Supplementary Data

Supplementary Table 1.1 *Percentages of supplements use*

Types of IPEDs	Percentage		
Caffeine	42.3%	Mineral Salt	8.3%
Whey protein	39.4%	Taurine	7.5%
Infusions	37.9%	Guaran	7.5%
Vitamins	37.2%	Ginseng	4.8%
Multivitamin supplement	29.3%	Glucosamine	3.5%
Omega 3 Fish Oil	25.3%	Beta alanine	2.3%
Creatine	23.1%	Nitric Oxide	1.9%
Amino Acids	22.1%	Ketones	1.2%
Multimineral supplement	16.3%	Pyruvate	0.8%
Herbal medicine	12.5%		
Turmeric	12.4%		
Green tea extract	10.9%		

Glutamate	10.3%
Carnitine	9.6%
Fish Oil	9.6%
Antioxidants	8.4%

Supplementary Table 1.2. *Percentages of off-label medications/drugs use*

Types of IPEDs	Percentage		
Ibuprofen	9.9%	Androgenic substances (e.g. steroids)	1.6%
Diuretics	6.5%	Beta Blockers	1.6%
Laxatives	6.3%	Hormones (e.g. pvz, EPO, insulin) or related (e.g. beta-2 agonists)	1.5%
Stimulants (e.g. amphetamine, modafinilas)	2.7%	Glucocorticoids	0.4%
Orlistat	1.8%		

Supplementary Table 1.3. *Sources of purchase (%)*

Sources of purchase	Percentage

Pharmacy	43.3%
Internet	41.0%
Specialised Food Store	29.9%
Food Store	28.1%
Other	3.5%
Black Market	1.0%

Supplementary Table 2. *ANOVA among the sports disciplines*

Sports	EAI	
Generic Workout	17.11±3.75	F = 8.11<br>p < .001
Walking	15.77±3.83	
Weight Lifting	18.02±3.70	
Running	17.79±3.46	
Yoga	16.65±3.86	
Fighting Sports	17.70±3.72	
Swimming	16.98±3.28	
Dance	17.41±3.64	

Martial Arts	17.76±3.38
Cycling	16.44±3.58
Ball Sports	17.62±4.49
Budo	17.36±3.04
Cross Fit	19.06±3.86

Sports	AAI	
Generic Workout	17.19±5.42	F = 6.98 p < .001
Walking	16.36±5.64	
Weight Lifting	18.35±5.93	
Running	16.40±5.57	
Yoga	16.18±4.76	
Fighting Sports	15.51±5.07	
Swimming	15.69±4.81	
Dance	18.09±6.16	
Martial Sports	15.75±5.23	
Cycling	14.29±4.41	
Ball Sports	16.49±4.95	
Budo	14.60±4.37	
Cross Fit	18.46±5.53	

NAG	16.65±5.59

Sports	SCS	
Generic Workout	30.87±5.96	F = 1.64<br>p < .052
Walking	31.11±6.10	
Weight Lifting	30.27±6.10	
Running	31.18±6.04	
Yoga	31.70±5.70	
Fighting Sports	31.40±5.53	
Swimming	31.42±5.94	
Dance	30.46±7.08	
Martial Arts	31.20±5.64	
Cycling	32.82±5.83	
Ball Sports	30.00±6.25	
Budo	31.33±5.57	
Cross Fit	31.49±5.54	
NAG	30.54±5.78	

Supplementary Table 3.1. *Tamhane's T2 method based post-hoc analysis on ANOVA (EAI)*

Multiple Comparisons

Dependent Variable: EAI

Tamhane's T2	(I) Sport	(J) Sport	Mean Difference (I-J)	Std. Error	Sig.	95% Confidence Interval Lower Bound	Upper Bound
	Running	Yoga	1.14*	.315	.027	.06	2.22
		Walking	2.02*	.284	.000	1.05	2.99
	Swimming	Cross Fit	-2.09*	.563	.020	-4.02	-.15
	Fighting Sports	Walking	1.93*	.359	.000	.70	3.16
	Martial Arts	Walking	1.99*	.400	.000	.62	3.37
	Budo	-	-	-	-	-	-
	Cycling	Weight Lift	-1.58*	.420	.017	-3.01	-.14
		Cross Fit	-2.62*	.595	.001	-4.66	-.58
	Ball Sports	Walking	1.85*	.471	.009	.23	2.48
	Generic Workout	Weight Lift	-.91*	.237	.011	-1.73	-.10
		Cross Fit	-1.96*	.484	.005	-3.62	-.30
		Walking	1.34*	.230	.000	.55	2.13
	Weight Lifting	Cycling	1.58*	.420	.017	.14	3.01
		Generic Workout	.91*	.237	.011	.10	1.73
		Yoga	1.37*	.304	.001	.33	2.41
		Walking	2.25*	.271	.000	1.32	3.18

Cross Fit	Swim	2.09*	.563	.020	.15	4.02
	Cycling	2.62*	.595	.001	.58	4.66
	Generic Workout	1.96*	.500	.001	.40	3.51
	Yoga	2.42*	.520	.000	.63	4.20
	Walking	3.30*	.502	.000	1.58	5.02
	Other	2.52*	.633	.007	.35	4.69
Mountain	Walking	2.12*	.536	.008	.28	3.96
Yoga	Run	-1.14*	.315	.027	-2.22	-.06
	Weight Lift	-1.37*	.304	.001	-2.41	-.33
	Cross Fit	-2.42*	.520	.000	-4.20	-.63
Walking	Run	-2.02*	.284	.000	-2.99	-1.05
	Fighting Sports	-1.93*	.359	.000	-3.16	-.70
	Martial Arts	-1.99*	.400	.000	-3.37	-.62
	Ball Sports	-1.85*	.471	.009	-3.46	-.23
	Generic Workout	-1.34*	.230	.000	-2.13	-.55
	Weight Lift	-2.25*	.271	.000	-3.18	-1.32
	Cross Fit	-3.30*	.502	.000	-5.02	-1.58
	Mountain	-2.12*	.536	.008	-3.96	-.28
	Dance	-1.64*	.377	.001	-2.93	-.35
Tennis	-	-	-	-	-	-

Other	Cross Fit	-2.52*	.633	.007	-4.69	-.35
Dance	Walking	1.64*	.377	.001	.35	2.93

Supplementary Table 3.2. *Tamhane's T2 method based post-hoc analysis on ANOVA (AAI)*

Multiple Comparisons

Dependent Variable: AAI

Tamhane's T2	(I) Sports	(J) Sports	Mean Difference (I-J)	Std. Error	Sig.	95% Confidence Interval	
						Lower Bound	Upper Bound
	Running	Weight Lift	-1.95*	.426	.001	-3.42	-.48
	Swimming	Weight Lift	-2.66*	.550	.000	-4.56	-.75
		Dance	-2.40*	.671	.034	-4.72	-.08
	Fighting Sports	Weight Lift	-2.84*	.535	.000	-4.69	-.99
		Cross Fit	-2.95*	.820	.031	-5.79	-.12
		Dance	-2.58*	.658	.010	-4.86	-.30
	Martial Arts	Weight Lift	-2.61*	.595	.002	-4.67	-.54

Budo	Generic Workout	-2.59*	.693	.019	-4.99	-.20
	Weight Lift	-3.75*	.724	.000	-6.25	-1.24
	Cross Fit	-3.86*	.954	.006	-7.16	-.56
	Dance	-3.49*	.820	.003	-6.33	-.65
Cycling	None	-2.36*	.633	.020	-4.55	-.17
	Generic Workout	-2.91*	.581	.000	-4.92	-.90
	Weight Lift	-4.06*	.618	.000	-6.20	-1.93
	Cross Fit	-4.18*	.876	.000	-7.21	-1.14
	Dance	-3.81*	.728	.000	-6.33	-1.29
Ball Sports	-	-	-	-	-	-
Generic Workout	Budo	2.59*	.693	.019	.20	4.99
	Cycling	2.91*	.581	.000	.90	4.92
Weight Lifting	None	1.70*	.431	.009	.21	3.20
	Run	1.95*	.426	.001	.48	3.42
	Swim	2.66*	.550	.000	.75	4.56
	Fighting Sports	2.84*	.535	.000	.99	4.69
	Martial Arts	2.61*	.595	.002	.54	4.67

	Budo	3.75*	.724	.000	1.24	6.25
	Cycling	4.06*	.618	.000	1.93	6.20
	Mountain	3.27*	.794	.004	.53	6.02
	Yoga	2.17*	.447	.000	.62	3.71
	Walking	1.99*	.400	.000	.61	3.37
	Tennis	3.38*	.963	.043	.04	6.71
Cross Fit	Fighting Sports	2.95*	.820	.031	.12	5.79
	Budo	3.86*	.954	.006	.56	7.16
	Cycling	4.18*	.876	.000	1.14	7.21
Mountain	Weight Lift	-3.27*	.794	.004	-6.02	-.53
Yoga	Weight Lift	-2.17*	.447	.000	-3.71	-.62
Walking	Weight Lift	-1.99*	.400	.000	-3.37	-.61
Tennis	Weight Lift	-3.38*	.963	.043	-6.71	-.04
Other	-	-	-	-	-	-
Dance	Swim	2.40*	.671	.034	.08	4.72
	Fighting Sports	2.58*	.658	.010	.30	4.86
	Budo	3.49*	.820	.003	.65	6.33

Cycling	3.81*	.728	.000	1.29	6.33

Supplementary Table 4. *Specification of each sports discipline*

	Generic Workout (N=769)	Others Exercise (N=1238)	
Usage of IPEDs	266(34.6%)	428(34.6%)	χ^2 =0.00 p = 0.993
Increase of smoking	50(31.8%)	73(35.6%)	χ^2 =0.56 p = 0.454
Increase of drinking	92(12.0%)	205(16.6%)	**χ^2 =7.95 p = 0.005**
History of addiction	50(6.5%)	92(7.4%)	χ^2 =0.623 p= .430
Worsening the addiction problem during physical distancing	15(30.0%)	27(29.3%)	χ^2 =0.007 p= .935

	Walking (N=387)	Other Exercise (N=1620)	
Usage of IPEDs	95(24.5%)	599(37.0%)	**χ^2 = 21.33 p <0.001**
Increase of smoking	22(31.0%)	101(34.7%)	χ^2 = 0.35 p =0.553
Increase of drinking	55(14.2%)	242(14.9%)	χ^2 = 0.13 p =0.718
History of addiction	39(10.1%)	103(6.4%)	**χ^2 =6.573 p= .010**

Worsening the addiction problem during physical distancing	15(38.5%)	27(26.2%)	χ^2 =2.037 p= .153

	Weight Lifting (N=355)	Others Exercise (N=1652)	
Usage of IPEDs	217(61.1%)	477(28.9%)	**χ^2 =134.37 p< 0.001**
Increase of smoking	9(17.3%)	114(36.8%)	**χ^2 =7.52 p= 0.006**
Increase of drinking	62(17.5%)	235(14.2)	χ^2 =2.43 p= 0.119
History of addiction	19(5.4%)	123(7.4%)	χ^2 =1.948 p= .163
Worsening the addiction problem during physical distancing	8(42.1%)	34(27.6%)	χ^2 =1.653 p= .199

	Running (N=301)	Others Exercise (N=1706)	
Usage of IPEDs	115(38.2%)	579(33.9%)	χ^2 = 2.06 p=0.151
Increase of smoking	18(35.3%)	105(33.8%)	χ^2 = 0.05 p=0.830
Increase of drinking	53(17.6%)	244(14.3%)	χ^2 = 2.22 p=0.136
History of addiction	22(7.3%)	120(7.0%)	χ^2 =0.029 p= .864
Worsening the addiction problem during physical distancing	5(22.7%)	37(30.8%)	χ^2 =0.586 p= .444

	Yoga (N=253)	Others Exercise (N=1754)	
Usage of IPEDs	79(31.2%)	615(35.1%)	χ^2 =1.44 p= 0.230
Increase of smoking	13(39.4%)	110(33.4%)	χ^2 =0.48 p= 0.491
Increase of drinking	38(15.0%)	259(14.8%)	χ^2 =0.01 p= 0.915
History of addiction	14(5.5%)	128(7.3%)	χ^2 =1.046 p= .306
Worsening the addiction problem during physical distancing	5(35.7%)	37(28.9%)	χ^2 =0.281 p= 596

	Fighting sports (N=146)	Others Exercise (N=1861)	
Usage of IPEDs	53(36.3%)	641(34.4%)	χ^2 =0.21 p = 0.650
Increase of smoking	13(61.9%)	110(32.3%)	**χ^2 =7.75 p = 0.005**
Increase of drinking	23(15.8%)	274(14.7%)	χ^2 =0.11 p = 0.736
History of addiction	11(7.5%)	131(7.0%)	χ^2 =0.050 p= .822
Worsening the addiction problem during physical distancing	5(54.5%)	37(28.2%)	χ^2 =1.443 p= .230

	Swimming (N=135)	Others Exercise (N=1872)	
Usage of IPEDs	42(31.1%)	652(34.8%)	χ^2 = 0.77 p = 0.380
Increase of smoking	4(21.1%)	119(34.7%)	χ^2 =1.49 p = 0.222

Increase of drinking	14(10.4%)	283(15.1%)	χ² =2.25 p = 0.134
History of addiction	6(4.4%)	136(7.3%)	χ² =1.524 p= .217
Worsening the addiction problem during physical distancing	3(50.0%)	39(28.7%)	χ² =1.254 p= 361

	**Dance** (N= 128)	**Others Exercise** (N= 1879)	
Usage of IPEDs	43(33.6%)	651(34.6%)	χ²=0.059 p= .809
Increase of smoking	5(27.8%)	118(34.3%)	χ²=0.325 p= .569
Increase of drinking	21(19.3%)	276(17.6%)	χ²=0.196 p= .658
History of addiction	10(7.8%)	132(7.0%)	χ²=0.113 p= 737
Worsening the addiction problem during physical distancing	3(30.0%)	39(29.5%)	χ²=0.001 p= 1.000

	**Martial Arts** (N=109)	**Others Exercise** (N=1898)	
Usage of IPEDs	36(33.0%)	658(34.7%)	χ² =0.12 p = 0.726
Increase of smoking	6(54.5%)	117(33.3%)	χ² =2.14 p = 0.144
Increase of drinking	14(12.8%)	283(14.9%)	χ² =0.35 = 0.555
History of addiction	7(6.4%)	135(7.1%)	χ² =0.075 p= .784
Worsening the addiction problem during physical distancing	3(42.9%)	39(28.9%)	χ² =0.623 p= .422

	Cycling (N=99)	Others Exercise (N=1908)	
Usage of IPEDs	26(26.3%)	668(35.0%)	χ^2 =3.18 p= 0.074
Increase of smoking	8(50.0%)	115(33.2%)	χ^2 =1.92 p = 0.166
Increase of drinking	14(14.1%)	283(14.8%)	χ^2 =0.04 p = 0.850
History of addiction	11(11.1%)	131(6.9%)	χ^2 =2.580 p= .108
Worsening the addiction problem during physical distancing	2(18.2%)	40(30.5%)	χ^2 =0.743 p= .507

	Ball Sports (N=73)	Others Exercise (N=1934)	
Usage of IPEDs	26(35.6%)	668(34.5%)	χ^2 =0.04 p = 0.849
Increase of smoking	5(31.3%)	118(34.1%)	χ^2 =0.06 p = 0.911
Increase of drinking	11(15.1%)	286(14.8%)	χ^2 =0.004 p = 0.814
History of addiction	5(6.8%)	137(7.1%)	χ^2 =0.006 p= .939
Worsening the addiction problem during physical distancing	0(0.0%)	42(30.7%)	χ^2 =2.177 p= .322

	Budo (N=67)	Others Exercise (N=1940)	
Usage of IPEDs	22(32.8%)	672(34.6%)	χ^2 =0.09 p = 0.760
Increase of smoking	3(60.0%)	120(33.6%)	χ^2 =1.53 p = 0.342
Increase of drinking	7(10.4%)	290(14.9%)	χ^2 =1.04 p = 0.308

History of addiction	4(6.0%)	138(7.1%)	χ^2 =0.129 p= 1.000
Worsening the addiction problem during physical distancing	1(25.0%)	41(29.7%)	χ^2 =0.041 p= 1.000

	**Cross Fit** (N= 63)	**Other Exercise** (N= 1944)	
Usage of IPEDs	**38(60.3%)**	**656(33.7%)**	**χ^2=19.047 p< .001**
Increase of smoking	6(50.0%)	117(33.4%)	χ^2=1.420 p= .233
Increase of drinking	14(26.4%)	283(17.4%)	χ^2=2.854 p= .091
History of addiction	6(9.5%)	136(7.0%)	χ^2 =0.593 p= .441
Worsening the addiction problem during physical distancing	0(0.0%)	42(30.9%)	χ^2 =2.631 p= .179

Abbreviations: EAI＝Exercise Addiction Inventory, AAI=Appearance Anxiety Inventory, SCS=Self-Compassion Scale

Note: χ^2 = chi square, t= Student's t-test

Supplementary Table 5. *Logistic regression models with specification of each activity*

		B	ES	Wald	df	Sig	Odd Ratio (OR)	Confidence Interval (CI)	
								Min	Max
**Usage of IPEDs (NAG)**	Age	.017	.017	1.052	1	.305	1.017	.984	1.052
	Gender	.265	.408	.422	1	.516	1.303	.586	2.899
	SCS total	.001	.032	.001	1	.981	1.001	.939	1.066
	AAI over the Cut Off	.3195	.431	.550	1	.458	1.376	.592	3.201
	Constant	-3.222	1.446	4.968	1	.026	.040		
**Usage of IPEDs (AG)**	Age	.001	.004	.064	1	.800	1.001	.993	1.010
	**Gender**	**-.488**	**.102**	**22.706**	**1**	**.000**	**.614**	**.502**	**.750**
	SCS total	.009	.009	1.170	1	.279	1.010	.992	1.027
	**EAI over the Cut Off**	**.800**	**.223**	**12.903**	**1**	**.000**	**2.226**	**1.438**	**3.444**
	**AAI over the Cut Off**	**.698**	**.126**	**30.824**	**1**	**.000**	**2.009**	**1.571**	**2.571**
	Constant	-1.839	.482	14.580	1	.000	.159		
**Usage of IPEDs (Generic Workout)**	Age	.009	.007	1.317	1	.251	1.009	.994	1.023
	**Gender**	**-.940**	**.179**	**27.426**	**1**	**.000**	**.391**	**.275**	**.555**
	SCS total	-.002	.014	.018	1	.892	.998	.970	1.027
	**EAI over the Cut Off**	**.716**	**.359**	**3.987**	**1**	**.046**	**2.047**	**1.013**	**4.135**
	**AAI over the Cut Off**	**.472**	**.208**	**5.136**	**1**	**.023**	**1.604**	**1.066**	**2.413**
	Constant	-.533	.776	.472	1	.492	.587		
**Usage of IPEDs (Walking)**	Age	.003	.009	.105	1	.746	1.003	.985	1.022
	Gender	.211	.287	.540	1	.462	1.235	.703	2.169
	SCS total	.020	.023	.724	1	.395	1.020	.975	1.067

	EAI over the Cut Off	.805	.710	1.284	1	.257	2.236	.556	8.991
	**AAI over the Cut off**	**.795**	**.327**	**5.908**	**1**	**.015**	**2.214**	**1.166**	**4.201**
	Constant	-4.016	1.397	8.260	1	.004	.018		
**Usage of IPEDs (Weight Lifting)**	Age	.013	.012	1.206	1	.272	1.013	.990	1.037
	**Gender**	**-.460**	**.231**	**3.963**	**1**	**.047**	**.631**	**.401**	**.993**
	SCS total	.015	.020	.589	1	.443	1.016	.976	1.056
	EAI over the Cut Off	.411	.450	.837	1	.360	1.509	.625	3.641
	**AAI over the Cut off**	**.744**	**.282**	**6.943**	**1**	**.008**	**2.104**	**1.210**	**3.658**
	Constant	-1.062	1.074	.977	1	.323	.346		
**Usage of IPEDs (Running)**	Age	.017	.012	2.130	1	.144	1.017	.994	1.041
	Gender	-.057	.248	.053	1	.818	.945	.581	1.536
	SCS total	.013	.022	.348	1	.555	1.013	.970	1.058
	EAI over the Cut Off	1.238	.709	3.051	1	.081	3.449	.860	13.832
	**AAI over the Cut off**	**.724**	**.335**	**4.670**	**1**	**.031**	**2.062**	**1.070**	**3.974**
	Constant	-3.515	1.268	7.685	1	.006	.030		
**Usage of IPEDs (Yoga)**	Age	.010	.013	.661	1	.416	1.010	.985	1.036
	Gender	-.559	.477	1.372	1	.241	.572	.224	1.457
	SCS total	.003	.027	.012	1	.912	1.003	.951	1.058
	**EAI over the Cut Off**	**2.283**	**.819**	**7.769**	**1**	**.005**	**9.805**	**1.969**	**48.824**
	AAI over the Cut off	.207	.428	.233	1	.629	1.230	.532	2.845
	Constant	-2.809	1.640	2.933	1	.087	.060		
**Usage of IPEDs (Fighting sports)**	Age	-.005	.017	.074	1	.786	.995	.963	1.029
	Gender	.003	.393	.000	1	.994	1.003	.465	2.165

	SCS total	.038	.036	1.101	1	.294	1.039	.967	1.116
	**EAI over the Cut Off**	**2.564**	**1.115**	**5.290**	**1**	**.021**	**12.984**	**1.461**	**115.410**
	**AAI over the Cut off**	**1.169**	**.528**	**4.908**	**1**	**.027**	**3.219**	**1.144**	**9.058**
	Constant	-5.671	2.088	7.376	1	.007	.003		
**Usage of IPEDs (Swimming)**	Age	-.008	.019	.197	1	.657	.992	.956	1.029
	Gender	-.027	.426	.004	1	.949	.973	.422	2.245
	SCS total	.011	.035	.095	1	.757	1.011	.943	1.084
	EAI over the Cut Off	21.815	22669.192	.000	1	.999	2.979E9	.000	.
	AAI over the Cut off	.917	.550	2.782	1	.095	2.501	.852	7.344
	Constant	-23.775	22669.192	.000	1	.999	.000		
**Usage of IPEDs (Dance)**	Age	.034	.018	3.573	1	.059	1.034	.999	1.071
	Gender	20.595	14023.111	.000	1	.999	8.798E8	.000	.
	SCS total	.006	.033	.030	1	.862	1.006	.943	1.073
	EAI over the Cut Off	.305	.767	.158	1	.691	1.357	.302	6.099
	AAI over the Cut off	**1.055**	**.491**	**4.611**	**1**	**.032**	**2.872**	**1.096**	**7.521**
	Constant	-44.733	28046.221	.000	1	.999	.000		
**Usage of IPEDs (Martial Arts)**	Age	.005	.020	.070	1	.792	1.005	.967	1.045
	Gender	.305	.468	.424	1	.515	1.357	.542	3.395
	SCS total	.040	.042	.895	1	.344	1.041	.958	1.131
	EAI over the Cut Off	1.592	1.210	1.730	1	.188	4.914	.458	52.691
	AAI over the Cut off	.933	.605	2.381	1	.123	2.542	.777	8.317
	Constant	-5.355	2.279	5.522	1	.019	.005		
**Usage of IPEDs**	Age	-.013	.020	.411	1	.521	.987	.948	1.027

**(Cycling)**	Gender	.209	.485	.185	1	.667	1.232	.476	3.185
	SCS total	-.039	.043	.821	1	.365	.962	.884	1.047
	EAI over the Cut Off	.546	1.625	.113	1	.737	1.727	.071	41.735
	AAI over the Cut off	.729	.827	.778	1	.378	2.073	.410	10.474
	Constant	-.931	2.662	.122	1	.727	.394		
**Usage of IPEDs (Ball Sports)**	Age	-.027	.038	.518	1	.472	.973	.903	1.048
	Gender	-.370	.591	.391	1	.532	.691	.217	2.201
	SCS total	-.018	.043	.165	1	.685	.983	.903	1.069
	EAI over the Cut Off	.216	.824	.069	1	.793	1.241	.247	6.243
	AAI over the Cut off	.526	.613	.735	1	.391	1.692	.508	5.632
	Constant	.242	2.171	.012	1	.911	1.274		
**Usage of IPEDs (Budo)**	Age	-.033	.025	1.800	1	.180	.967	.922	1.015
	Gender	.294	.567	.269	1	.604	1.342	.442	4.080
	SCS total	.060	.056	1.183	1	.277	1.062	.953	1.185
	EAI over the Cut Off	22.561	40192.886	.000	1	1.000	6.284E9	.000	.
	AAI over the Cut off	.344	.856	.161	1	.688	1.411	.263	7.557
	Constant	-24.756	40192.886	.000	1	1.000	.000		
**Usage of IPEDs (Cross Fit)**	Age	.013	.037	.113	1	.737	1.013	.941	1.090
	Gender	-.694	.596	1.356	1	.244	.499	.155	1.607
	SCS total	-.005	.055	.008	1	.931	.995	.893	1.109
	EAI over the Cut Off	.767	1.227	.391	1	.532	2.154	.194	23.868
	AAI over the Cut off	.454	.653	.483	1	.487	1.574	.438	5.656
	Constant	-.048	2.986	.000	1	.987	.953		

Gender: “1” – male, “2” – female; EAI over the Cut Off : “0” < 24 , “1”≥24; AAI over the Cut Off : “0” < 21 , “1”≥21.

Supplementary Figure 1. The figure legends are required to have the same font as the main text, 12 point normal Times New Roman, single spaced. Please use a single paragraph for each legend and prepare the figures keeping in mind the PDF layout.
